# An update on diabetic kidney disease, oxidative stress and antioxidant agents

**DOI:** 10.15171/jrip.2017.30

**Published:** 2017-01-20

**Authors:** Leila Mahmoodnia, Esmat Aghadavod, Sara Beigrezaei, Mahmoud Rafieian-Kopaei

**Affiliations:** ^1^Department of Internal Medicine, Shahrekord University of Medical Sciences, Shahrekord, Iran; ^2^Research Center for Biochemistry and Nutrition in Metabolic Diseases, Kashan University of Medical Sciences, Kashan, Iran; ^3^School of Nutrition & Food Sciences, Isfahan University of Medical Sciences, Isfahan, Iran; ^4^Medical Plants Research Center, Shahrekord University of Medical Sciences, Shahrekord, Iran

**Keywords:** Antioxidant, Oxidative stress, Diabetes mellitus, Kidney disease

## Abstract

Diabetes mellitus is a metabolic disease that is defined by relative or absolute deficiency of insulin secretion. Diabetic kidney disease seems to be one of the most frequent complications of diabetes mellitus. Based on evidence, increased free-radical formation and/or diminished antioxidant defenses induce oxidative stress that is implicated in the pathogenesis of diabetic kidney disease. It is evident that diabetic state induces oxidative stress through different signaling pathways as well as reactive oxygen species (ROS) formation that attributes to the activation of various downstream signaling cascade leading to structural the way to structural and functional changes in kidney.

**Implication for health policy/practice/research/medical education:**

It is evident that diabetic state induces oxidative stress through different signaling pathways as well as reactive oxygen species (ROS) formation that attributes to the activation of various downstream signaling cascade leading to the way to structural and functional changes in kidney.

## Introduction

Diabetes mellitus is a metabolic disease that is defined by relative or absolute deficiency of insulin secretion. Today, diabetes is known to be one of the primary causes of mortality and morbidity in the world (1-3). Complications of disease progression include retinopathy, nephropathy, cardiomyopathy, hepatopathy, and neuropathy. Diabetes is generally comprised of several subcategories such as type 1 diabetes mellitus which demonstrated by absolute insulin deficiency caused by cell-specific autoimmune destruction of the insulin producing beta cells in the pancreas. Type 2 diabetes which is occurred as a result of the weakness of beta cells to compensate for insulin secretion or selective loss of pancreatic beta cells due to viral infections or toxic injury leading to insulin insufficiency (4). Diabetic kidney disease is one of the critical problems of diabetes mellitus which its prevalence has been increasing in worldwide (5). Generally, chronic hyperglycemia increases oxidative stress factors followed by modifying the structure and function of proteins and lipids significantly. It also induces glycoxidation and peroxidation. Thus, hyperglycemia results to auto-oxidation of glucose, glycation of proteins and activation of polyol mechanism. Hyperglycemia-induced oxidative stress has been singled out as one of the main links between diabetes and diabetic complications. Therefore; hyperglycemia induces autoxidation of glucose and glycosylation of proteins by generation of free radicals thus, increases the reactive oxygen species (ROS) accompanied by a reduction in antioxidant activity which leads to the occurrence of oxidative stress. These can cause endothelial dysfunction, insulin resistance, and alterations in the proportion and functions of pancreatic 𝛽 cells and ultimately leads to diabetic microvascular and macrovascular complications (6,7). Based on evidence, ROS induces several cellular signaling cascades which in turn promote transcription of stress-related genes and development of diabetic complications including nuclear factor kappa-light-chain-enhancer of activated B cells (NF-𝜅B), an activated nuclear transcription factor by an elevation in ROS. This phenomenon ultimately results in transcription of pro-inflammatory proteins. Increase in ROS can activate pro-inflammatory chemokines, tumor necrosis factor (TNF-𝛼), macrophage chemotactic proteins (MCP-1), and interleukins (IL-1β and 6) that have been implicated in the progression of diabetes to diabetic complications (8). Likewise, hyperglycemia can induce ROS elevation and can lead to activation of apoptosis signaling pathways in tissues by Bax-caspase pathway. This in turn can lead to reduction of electrochemical gradient through the mitochondrial membrane causing a leakage of mitochondrial cytochrome C into cytoplasm. This process also may activate apoptosis (9). This review article aimed to discuss the pathophysiological mechanisms of renin-angiotensin system (RAS) inhibition by herbal plants and update the diabetic nephropathy, oxidative stress and antioxidant agents.

## Materials and Methods

For this review, we used a variety of sources by searching through Web of Science, PubMed, EMBASE, Scopus and directory of open access journals (DOAJ). The search was performed using combinations of the following key words and or their equivalents; diabetic nephropathy, reactive oxygen species, diabetic kidney disease, chronic kidney disease, microalbuminuria and advanced glycation end products.

## Diabetic kidney disease

Diabetic kidney disease seems to be one of the most frequent complications of diabetes mellitus. Based on evidence, increased free-radical formation and/or diminished antioxidant defenses induce oxidative stress that is implicated in the pathogenesis of diabetic kidney disease (10-12). Antioxidants include enzymatic antioxidants such as superoxide dismutase (SOD), catalase (CAT), glutathione S-transferase (GST), glutathione peroxidase (GPx), and non-enzymatic antioxidants such as reduced glutathione (GSH), uric acid, carotenoids, flavonoids, lipoic acid, and vitamins A, C, and E. Superoxide anion (∙O2−) dismutates are manufactured to produce water by CAT and GPx. Enzymatic antioxidant of GST converts reactive electrophilic species to hydrophilic forms then are conjugated by GSH and easily excreted. Non-enzymatic antioxidants like vitamins C and E and lipoic acid are involved in the termination of the lipid peroxidation process (13,14).

## Oxidative stress and diabetes

Generally, oxidative stress happens when the proportion of production of ROS into the cell exceeds their normal rates, which leading to cellular and tissue damage (15). Therefore, imbalance of oxidants/antioxidant ratio causes an alteration in the normal redox signaling of the cell that induces impairment in several pathways of the cell’s metabolism. ROS, free radicals or non-free radical compounds, can be either useful or harmful to the cells. Free radicals of ROS include superoxide (∙O_2_−), hydroxyl (∙HO), peroxyl (∙RO_2_−), hydroperoxyl (∙HRO_2_−), and non-radical of ROS include hydrogen peroxide (H_2_O_2_) and hydrochlorous acid (HOCl). Hyperglycemia-induced oxidative stress can inhibit the secretion of insulin in pancreatic beta cell through the activation of an uncoupling protein-2 (UCP-2) which lowers the ATP/ADP ratio by leaking protons in the β cell. Also, ROS can leak into cell membranes and damage pancreatic β cells. Some researchers showed overproduction of free radicals such as superoxide anion in β cells leading to activation of stress-signaling processes which are able to induce downstream effectors such as NF-𝜅B which may lead to β cell apoptosis and dysfunction in the end reducing insulin secretion. Also, high concentrations of hydrogen peroxide directly with the phosphatidylinositol-3-kinase dependent pathway induce insulin signaling leading to insulin resistance prior to the onset of diabetes. Some reports have detected that in presence of hyperglycemia, hyperlipidemia can generate more ROS imposing β cell dysfunction. Likewise, high free fatty acids affect ROS overproduction leading to mitochondrial DNA damage and pancreatic β cell dysfunction (16).

## Oxidative stress and diabetic nephropathy

Diabetic kidney disease is considered as one of the major micro-vascular problems of diabetes mellitus and has become the most general single cause of end-stage kidney disease. It is defined traditionally by kidney morphological and functional modifications like; glomerular hyperfiltration, glomerular and kidney hypertrophy, increased urinary albumin excretion, increased glomerular basement membrane (GBM) thickness, and mesangial expansion and also accumulation of extracellular proteins comprising laminin, collagens and fibronectin (17,18). It is now clear that multiple pathways are involved in the pathogenesis of diabetic kidney disease like; hexosamine pathway, protein kinase C (PKC) pathway, formation of advanced glycation end products (AGEs), polyol pathway, growth factors, cytokines and free radicals. It should be noted that all these paths are induced by imbalance between oxidative stress and antioxidant elements (19).

Oxidative stress is a poisonous phenomenon within the body that principally is illustrated by disparity of cellular oxidation/reduction levels. On the other hand, it occurs when reduction of the normal antioxidant defense system of the human body such as CAT, GPx, SOD and glutathione reductase (GR) is counteracted. High glucose level, either due to resistance of cells to insulin or lack of insulin synthesis, plays an important role in production of ROS through the activation of various cellular responses thereby leading to deleterious effects like diabetic kidney disease. There are several factors which are involved in generation of oxidative stress during diabetes such as peroxyl, hydroxyl, superoxide and many other reactive compounds hydrogen peroxide (H_2_O_2_), nitric oxide (NO), nitrous oxide (HNO2), hydrochlorous acid (HOCl) and nitrogen dioxide (NO_2_). Also, the evidence show that oxidative stress during diabetic kidney disease eventually results to the knocking down of normal kidney homeostasis and its proper functioning. It is well established that augmented ROS construction in kidneys of both type 1 and type 2 DM can be affected individuals through both enzymatic and non-enzymatic pathways (18).

On the evidence, high glucose acts by the activation of PKC in diabetic glomeruli via de novo synthesis of diacylglycerol (DAG). Then, PKC creates ROS which finally cause activation of PKC thus causing enhancement of mesangial expansion, GBM thickening and dysfunction of endothelial cells running to diabetic kidney disease. Furthermore, high glucose levels induce isoforms of nicotinamide adenine dinucleotide phosphate oxidases (NOXs), especially NOX-4 which results to endothelial dysfunction, inflammation and consequently apoptosis. Also, the others pathways of glucose auto-oxidation such as increased formation of AGEs, polyol pathway flux and glycation of proteins are involved in causing direct and indirect kidney injury by producing extensive proportion of ROS. Accordingly, diabetic kidney disease is also associated with an intensification in RAS activity, as well as aldosterone production which play an important and direct role in the creation and activation of ROS resulting to kidney injury. On the other hand, high glucose level leads to the induction of transforming growth factor-(TGF-β) presentation in the glomerular and tubulointerstitial compartments that enhances the level of TGF-β leading to kidney enlargement, glomerulosclerosis and tubulointerstitial fibrosis in diabetic kidney disease (18-20). Under oxidative stress situation, vascular endothelial growth factor (VEGF), a protein secreted by the podocytes and the mesangial renal cells that plays a role in the extension of diabetic kidney disease. In fact, VEGF is increased by the activity of hypoxia-inducible factor (HIF-1α) and augmented level of angiotensin II. Some literatures reported that VEGF may interfere with the pathway of phosphoinositide 3-kinase/protein kinase B (PI3K/PKB) and presentation of endothelial nitric oxide synthase (eNOS). Therefore; VEGF indirectly stimulates the level of intracellular ROS by provocation the generation of peroxynitrite (ONOO−). Consequently, ROS production by various pathways results to the activation of downstream molecules like NF-κB, TGF-β and p38 mitogen activated protein kinases (p38 MAPK), pathway ultimately results to nephropathic condition by activating an array of diverging signaling cascades (20).

## Antioxidant strategies in diabetic nephropathy

Antioxidant compounds are important for two main reasons. First, they can reduce the harmful effects of free radicals not only by preventing their formation but also by scavenging and inactivating free radicals or improving natural defense systems by inducing the activities of antioxidant enzymes and regenerating other proteins involved in antioxidant pathways. There are several strategies employed in the use of different antioxidants to contrast diabetic nephropathy. The choice of strategy depends on the type, structure, and concentration of the antioxidants. In addition, the stage, severity and prevalence of the disease are very important (21,22).

## Vitamin E and diabetic nephropathy

Vitamin E, a lipid-soluble vitamin, includes the stereoisomers a-tocopherol, b-tocopherol, g-tocopherol, d–tocopherol and d–tocotrienol that tocopherol and tocotrienol stereoisomers and their metabolites appear to have potent biological properties. Generally, vitamin E detoxifies free radicals directly and also interacts with recycling processes to create reduced forms of A and C vitamins. Vitamin E also has a number of biological activities such as immune stimulatory and preventive activity of genetic changes by inhibition of DNA damage. Vitamin E primarily serves as a chain-breaking antioxidant to prevent lipid peroxidation. Some literatures have shown that tocopherol can modulate cell signaling pathways by inhibiting PKC. Tocopherols also have gene regulatory functions because they potentially serve as ligands for the transcription factor peroxisome proliferators-activated receptor-γ (PPAR-γ). The tocopherol concentrations of plasma are lower in diabetics compared to controls and appear to be even lower in diabetics with complications like microangiopathy than diabetics without complications (23).

## Q10 and diabetic nephropathy

Coenzyme Q10 (COQ10), an endogenous lipid-soluble micronutrient, is found in most living cells in the body that is a key component in the electron transport chain of the mitochondria, serving in the process of adenosine triphosphate (ATP) synthesis. Also, CoQ10 is a powerful antioxidant that scavenges free radicals and provides protection to cells in oxidative stress condition. Based on evidence, individuals with diabetic kidney disease often have deficiency in CoQ10 plasma levels compared to normal healthy subjects. Because of its antioxidant property, it is accepted that CoQ10 deficiency may impair the body’s defensive mechanisms against oxidative stress induced by hyperglycemia (24). Therefore, it is recommended that CoQ10 plays an important role in the pathogenesis of type 2 diabetic mellitus. Also, it is suggested that CoQ10 deficiency is a precipitating factor for diabetic kidney disease (25). Researchers have explored the effects of CoQ10 on glycemic control among individuals with type 2 diabetic mellitus and they investigated that daily supplementation of CoQ10 (200 mg/d) for 12 weeks effectively decreased HbA1c levels among patients with uncomplicated type 2 diabetic mellitus and dyslipidemia. In addition, the researchers suggested that CoQ10 provides protection to β cells through its antioxidant property, down-regulation of insulin receptors to improve insulin action and provides protection to the kidneys in the event of diabetic nephropathy. It is clear that CoQ10 is a scavenger of ROS and provides protection to cells, especially mitochondria from oxidative damage condition (26).

## Alpha-lipoic acid and diabetic nephropathy

Alpha-lipoic acid (ALA), one of the most successful antioxidants in clinical trials, is the only antioxidant capable of dissolving in both water and fats. It can be biosynthesized in plants and animals then metabolized to dihydrolipoic acid (DHLA) upon uptake into cells. Based on evidence, ALA and DHLA are powerful free radical scavengers that are also involved in the regeneration of vitamins C and E and oxidized glutathione within the cells. Also, ALA is a cofactor for a number of mitochondrial enzymes that can decrease lipid peroxidation, reduce oxidative stress, and improve blood flow in kidney. Some studies have shown that ALA reduces oxidative stress by inhibiting hexosamine and AGEs pathways (27).

## Vitamin D and diabetic nephropathy

There is an inverse relationship between serum 25-hydroxyvitamin D [25(OH) D_3_] levels or prevalence of diabetes mellitus and its complications. Also, some previous studies showed an improvement of diabetic kidney disease symptoms after vitamin D supplementation. It has been demonstrated that vitamin D might reverse the progression of diabetic kidney disease for instance improving glucose metabolism, minimizing RAS activation, and finally reducing fibrosis (28). A study showed that vitamin D receptor was highly expressed in the kidney. Hence the kidney could be considered a classic vitamin D target organ. Also, an inverse correlation was reported between the prevalence of albuminuria and serum 25(OH)D_3_ concentration. The mechanism for relationship between vitamin D and diabetic kidney disease remains unclear however various animal studies have shown that knockout of the vitamin D receptor in diabetic mice is accompanying by severe albuminuria and glomerulosclerosis. Therefore, vitamin D might slow down diabetic kidney disease progression by delaying destruction of b-islet cells, enhancing insulin secretion, and consequently assisting in glucose metabolism (29,30).

## Flavonoids and diabetic nephropathy

Flavonoids, the largest and the most important group of polyphenolic compounds in plants, are found in fruits, vegetables, grains, bark, roots, stems, flowers, tea, and wine. Flavonoids are made up of several subclasses that can scavenge free radicals and chelate metals for example; pro-anthocyanidin and luteolin have been shown to possess antioxidant activities which protect against diabetic kidney disease (31).

## Conclusion

The advantage of knowledge of powerful antioxidants is finding the modalities for greater insight in the treatment of diabetic kidney disease. In this review, we focused on understanding of diabetes induced oxidative stress through different signaling pathways as well as ROS formation that attributes to the activation of various downstream signaling cascade leading the way to structural and functional changes in kidney. We discussed that the administration of the anti-oxidative agents was able to restore the antioxidant defense system thereby preventing ROS mediated injuries.

## Authors’ contribution

SB and LM searched the articles. EA prepared primary draft. SB and LM performed first edition. MRK did final edition. All authors wrote and signed the final paper.

## Conflicts of interest

The authors declared no competing interests.

## Ethical considerations

Ethical issues (including plagiarism, double publication) have been completely observed by the authors.

## Funding/Support

The authors received no financial support for the research, authorship, and/or publication of this article.
